# Pituitary Gland Functional Connectivity and BMI

**DOI:** 10.3389/fnins.2019.00120

**Published:** 2019-03-01

**Authors:** Paige Rucker, Toshikazu Ikuta

**Affiliations:** ^1^School of Medicine, The University of Mississippi Medical Center, Jackson, MS, United States; ^2^Department of Communication Sciences and Disorders, The University of Mississippi, Oxford, MS, United States

**Keywords:** pituitary gland, functional connectivity, resting state fMRI, gustatory cortex, caudate head

## Abstract

The pituitary gland (PG) influences body weight through hormonal releases; however, the relation between body weight and PG’s co-activities with other brain regions remains unclear. Here, we aimed to identify (1) the functional connectivity of the PG and (2) PG functional connectivity associated with body mass index by examining resting state functional magnetic resonance imaging data. Using enhanced Nathan Kline Institute-Rockland Sample, PG functional connectivity of 494 individuals was analyzed to assess in voxel-wise fashion. A negative association was found between BMI and PG functional connectivity with the orbitofrontal cortex, hippocampus, putamen, and temporal lobe. Our results show PG dysconnectivity to these regions is associated with higher BMI and implicate that the connectivity between these dopaminergic regions and PG may be associated with body weight maintenance through feeding behavior and growth.

## Introduction

The hypothalamic-pituitary-adrenal (HPA) axis including the pituitary gland (PG) has been shown to be associated with obesity (Pasquali et al., 2006). In obesity, responses to growth hormone releasing factor are impaired (Williams et al., 1984). Chronic stress has been implicated to promote obesity through the HPA axis (Bose et al., 2009). Genetic variation in leptin receptors also shows association with obesity (Clement et al., 1998). The PG synthesizes and secretes hormones that regulate body weight, including somatotropins and oxytocin; however, the brain regions responsible for associating the PG activity and obesity are not clearly known.

Functional connectivity analysis using resting state fMRI (Fox and Raichle, 2007; Biswal et al., 2010) has provided newer insights into brain coactivity and obesity. The default mode network (DMN) and temporal lobe network have been found to be associated with obesity (Kullmann et al., 2012). In obesity, the precuneus shows an increased functional connectivity in the DMN while that of the anterior cingulate cortex is decreased. The insular cortex shows reduced connectivity in the temporal lobe network. Dysconnectivity between the hypothalamus and left insula has been found in obesity (Wijngaarden et al., 2015). After 48 h of fasting, connectivity of the hypothalamus to the dorsal anterior cingulate cortex increases in lean populations and decreases in the obese populations, suggesting a differential influence of obesity to functional connectivity of the hypothalamus (Wijngaarden et al., 2015). The putamen has been found to have elevated functional connectivity in obesity, while cognitive processing speed was negatively associated with the connectivity of the putamen to the salience network, suggesting an altered processing of salience detection (García-García et al., 2013). Obesity-preventive eating tendency has been found to be associated with caudate-precuneus functional connectivity (Nakamura and Ikuta, 2017). Caudate-precuneus connectivity inversely predicts the personal characteristics of avoiding obesity-inducing behaviors, suggesting the functional connectivity signature of avoiding causes of obesity independent of current body weight status. Surgical and behavioral weight loss has been found to differentially influence functional connectivity (Lepping et al., 2015). Despite its strong relationships to obesity, the influence of functional connectivity of the PG to body weight has not been yet studied.

Resting state functional connectivity of the PG is minimally studied, although functional connectivity is associated with adrenocorticotropic hormone (ACTH) levels. Cortisol concentrations have been found to predict interhemispheric connectivity, and ACTH concentrations were shown to be associated with the subcallosal anterior cingulate cortex (Kiem et al., 2013). Nonetheless, the resting state PG connectivity remains unclear. In this study, we (1) examined the resting state functional connectivity of the PG and (2) aimed to isolate resting state functional connectivity of the PG associated with BMI.

## Materials and Methods

### Data Acquisition

The MRI images, the clinical data, and the demographic data of the enhanced Nathan Kline Institute-Rockland Sample (Nooner et al., 2012) were obtained from Collaborative Informatics and Neuroimaging Suite (Biswal et al., 2010). This data subset consisted of 494 individuals without known neurological preconditions (such as stroke, tumor, and traumatic brain damage) and MRI counter indications (43.46 ± 20.81 years old), 310 females and 184 males, six Native Americans, 25 Asians, 102 Black or African Americans, two Native Hawaiians, 346 Caucasians, and 13 other races, with the mean BMI of 27.32 ± 6.35 (between 15.29 and 56.28), for whom resting state and structural data were both available. Participants in the sample were recruited from Rockland County, NY, whose demographics represent the United States (Nooner et al., 2012). The subjects with known pituitary conditions (such as pituitary tumor) were not included in the analysis data.

Resting state echo planar image (EPI) volumes had 64 slices of 2 mm 112 × 112 matrix with 2 mm thickness (voxel size = 2 mm × 2 mm × 2 mm), FOV = 224 mm, with repetition time (TR) of 1400 ms and echo time (TE) of 30 ms. A total of 404 volumes (∼10 min) were used in the analysis. High-resolution structural T1 volume was acquired as 176 sagittal slices of with 1 mm thickness (voxel size = 1 mm × 1 mm ×1 mm, TR = 1900 ms and TE = 2.52 ms, FOV = 256).

### Data Processing

Data processing followed previous publication (Kiparizoska and Ikuta, 2017). Data preprocessing and statistical analyses were conducted using FMRIB Software Library (FSL,) as well as Analysis of Functional NeuroImages (AFNI). The anatomical volume for each subject was skull stripped, segmented (gray matter, white matter, and CSF), and registered to the MNI 2 mm standard brain. First four EPI volumes were removed. Transient signal spikes were removed by de-spiking interpolation. To correct head motion, the volumes were linearly registered to the then first volume, through which six motion parameters and displacement distance between two consecutive volumes were estimated. The first volume is registered to the standard MNI152 2 mm brain. Through this registration, 12 affine parameters were created between rs-fMRI volume and MNI152 2 mm space, so that the processed EPI volume can later be registered to the MNI space. Each of the resting state volumes was regressed by white matter and cerebrospinal fluid signal fluctuations as well as the six motion parameters. After smoothing with a 6 mm FWHM Gaussian kernel, the volumes were resampled, spatially transformed and aligned to the MNI 2 mm standard brain space. To perform scrubbing where the volumes with excess motion are removed, as a displacement distance between two EPI volumes, the root mean square deviation was calculated from motion correction parameters, at an *r* = 40 mm spherical surface using FSL’s rmsdiff tool (Power et al., 2012, 2015). Volumes whose displacement distance exceeded the threshold (0.3 mm) were removed (*scrubbed*) from further statistical analyses (Siegel et al., 2014).

The PG ROI was manually defined in the MNI 2 mm space centered approximately at [MNI: 0, 2, -32] (Figure 2: Red), following previous MRI literature (Klomp et al., 2012). Data were excluded if the PG was located outside of the acquisition. Voxel-wise connectivity analysis was conducted in each individual brain. The time course was spatially averaged within the PG ROI that was registered to the EPI space so that correlations could be tested between the ROI and each individual voxel across the brain. The *Z*-scores representing the correlations between the ROI and a voxel were used for group level analysis after registration to the MNI 2 mm brain space.

In order to elucidate the regions which showed functional connectivity and dysconnectivity, one-sample *t* test was conducted assessing correlation (positive connectivity) and anti-correlation (negative connectivity) to the PG. Using *randomize* script in FSL, contrast images were estimated with cluster threshold of *Z* > 3.72. The association between BMI and whole brain functional connectivity to the PG was tested in a voxel-wise fashion using *randomize* script in FSL, taking age as a covariate. Contrast images were estimated with voxel-wise threshold of *p* < 0.05 (family wise error corrected), and minimum cluster size of 10 voxels.

## Results

**FIGURE 1:**
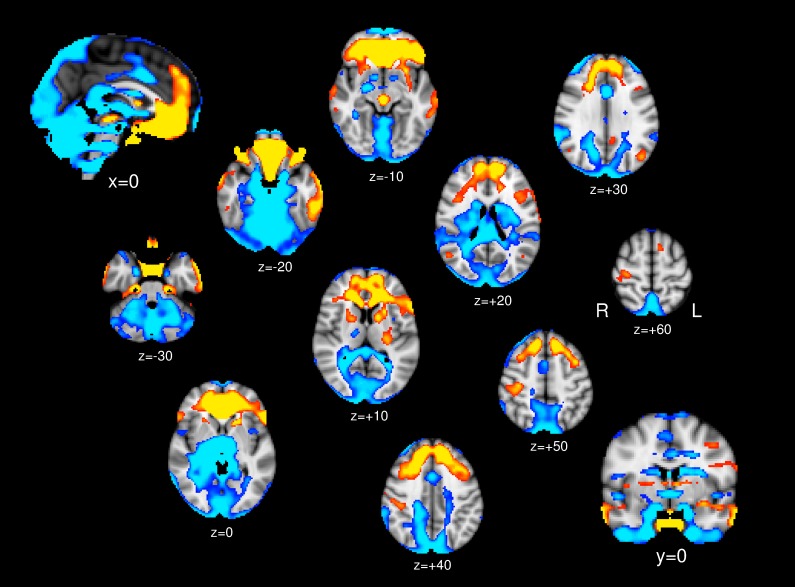
One-sample *t* test; Brain regions show significant positive connectivity (yellow/red) and negative connectivity (blue) to the PG.

In one-sample *t* test, in addition to the hypothalamus, the ventral and medial prefrontal cortex, inferior temporal gyrus, postcentral gyrus, insular cortex, parahippocampal gyrus, putamen, caudate head, and midbrain (periaqueductal gray), bilaterally showed positive connectivity with the PG (yellow/red in Figure 1). The dorsolateral prefrontal, parietal, occipital, and anterior cingulate cortices, hippocampus, caudate body, thalamus, pons medulla, and cerebellum showed bilateral negative connectivity (blue in Figure 1).

**FIGURE 2:**
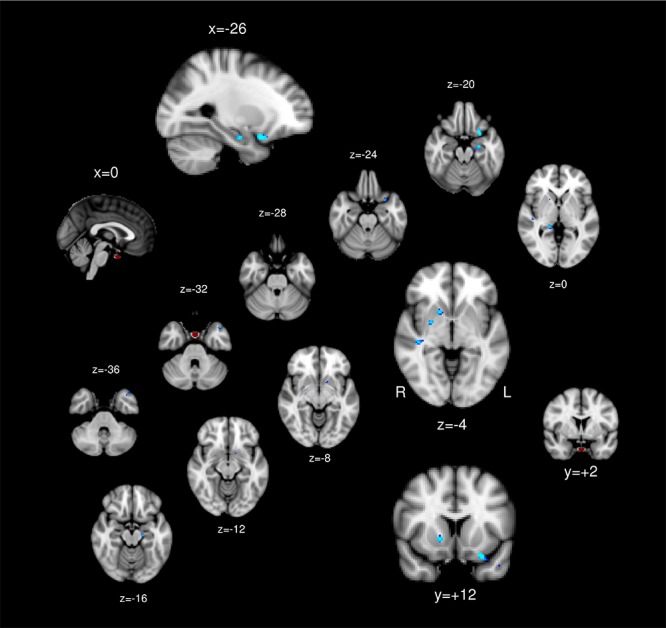
The pituitary ROI (red) and regions whose pituitary connectivity showed negative association with BMI (blue).

No regions showed positive association between the PG functional connectivity and BMI. The left orbitofrontal cortex, bilateral hippocampus, bilateral putamen, and right superior temporal gyrus showed functional connectivity with the PG inversely associated with BMI (Table 1 and Figure 2).

**Table 1:** Regions whose PG connectivity showed negative association with BMI.

	Voxels	Peak *p* (corrected)	Cluster *p* (corrected)	MNI coordinates			Cluster Region
				x	y	z	
1	45	0.0002	0.015	-26	12	-20	Left Orbitofrontal Cortex
2	41	0.0012	0.019	-24	-12	-20	Left Hippocampus
3	33	0.0064	0.046	40	-26	-4	Right Superior Temporal Gyrus
4	23	0.0024	0.019	16	10	-4	Right Putamen
5	22	0.006	0.022	26	-2	-4	Right Putamen/Pallidum
6	21	0.013	0.030	-44	16	-30	Left Temporal Pole
7	14	0.009	0.024	16	-36	0	Right Hippocampus
8	10	0.023	0.035	-14	8	-10	Left Putamen


## Discussion

This study aimed to identify (1) the functional connectivity of the PG and (2) brain regions whose functional connectivity to the PG shows associations with Body Mass Index. In one-sample *T*-test, the hypothalamus showed positive associations with the PG. This conforms to their close relationship classically know as a part of the HPA axis, which is known to be associated with obesity (Chalew et al., 1995). In this current study, however, the hypothalamus-PG functional connectivity did not show significant association with BMI.

Given their close relationship, we conducted an ROI-ROI connectivity analysis between the hypothalamus and PG. To assess the association between the BMI and hypothalamus-PG connectivity, a multiple linear regression was calculated to predict the hypothalamus-PG connectivity based on BMI, age, and sex. The regression was not significant, suggesting that the connectivity between hypothalamus-PG connectivity is constant across BMI, while PG and hypothalamus connectivity to the other parts of the brain identified in our voxel-wise analysis are associated with BMI. That is, the PG and hypothalamus are, similarly, influenced by the rest of the brain, resulting in the hypothalamus-PG connectivity remaining constant across BMI. Since the hypothalamus and PG are strongly positively connected, it is expected that the functional connectivity of the hypothalamus would show similar pattern with the PG in its association with BMI.

The orbitofrontal cortex (OFC) showed positive functional connectivity with the PG and inverse association with the BMI in its connectivity to the PG. It is implicated that lesser connectivity between the PG and OFC is associated with BMI. The OFC has been shown to be associated with obesity, as well as being responsible for taste and flavor processing (Small et al., 2007) and food choice (Cohen et al., 2011). Reduced OFC gray matter volume has been found in both adolescent (Ross et al., 2015) and midlife obesity (Driscoll et al., 2011). The OFC volume has been found to predict the risk for obesity (Smucny et al., 2012). More specifically, the OFC has been implicated in its role in preventing overeating. The OFC has been shown to be responsible for disinhibition of eating (Maayan et al., 2012), implicating its importance in preventing obesity. OFC volume has also been found to be associated with reward response in obesity (Shott et al., 2015). Reduced connectivity between the PG and OFC in a more obese population may suggest downregulated control of the OFC over PG.

The putamen showed inverse association between PG connectivity and BMI. That is, lesser PG-putamen connectivity is implicated in higher BMI. The association between pituitary-putamen dysconnectivity and BMI may be also accounted for by its role in the reward processing since the putamen and PG are both rich in dopamine D2 receptors. Striatal D2 receptors were shown to be reduced in individuals with obesity (Wang et al., 2004) and deficits of D2 receptor availability predicts future weight gain (Michaelides et al., 2012). Antipsychotics, whose pharmacological mechanism is represented by D2 antagonism, is widely known to induce weight gain (Correll et al., 2011). A D2 agonist bromocriptine has been found to counteract obesity (Kok et al., 2006). On the genetic level, polymorphisms in the D2 receptor gene (*DRD2*) have been implicated in its association to obesity (Nisoli et al., 2007; Ariza et al., 2012), as well as the weight gain response to antipsychotics (Lencz et al., 2010). The D2R profiles in the PG also show association with obesity. Disrupting D2 receptors in the pituitary lactotropes results in weight gain (Perez Millan et al., 2014). Dopaminergic modulations in the PG and striatum are both implicated in their association with BMI.

The PG receives dopaminergic projections from the hypothalamic arcuate nucleus as the terminal of the tuberoinfundibular pathway. Although this may partially account for their lack of BMI-differentiated association, it does not account for the associations with the striatal regions which are independently dopaminergic as the terminal of mesolimbic dopaminergic pathway originating in the substantia niagra. While the striatum and PG are independently dopaminergic in their primary source of their dopaminergic afferents, prolactin has been shown to upregulate both tuberoinfundibular and striatal dopamine neurons. While dopamine is inhibitory against secretion of prolactin (Ben-Jonathan, 1985; Liang et al., 2014), prolactin has been found to promote dopamine discharge in the tuberoinfundibular pathway (Lyons et al., 2012), forming a loop to regulate serum prolactin. In the striatum, rat prolactin increases dopamine turnover in the striatum (Fuxe et al., 1977). Prolonged intake of excessive sucrose has been found to upregulate striatal prolactin, thus showing implication to induce compulsive eating behavior (Ahmed et al., 2014). The prolactin-mediated dopaminergic response may be the underlying factor in the association between pituitary-caudate/NAcc functional dysconnectivity and BMI, whereby dopaminergic neurons in the PG and striatum are downregulated by prolactin.

It has to be noted that prolactin would not be the only mechanism that could both interact with the PG and striatum in the complex HPA axis. A voxel-based morphometric study showed a reduced volume of the putamen in obesity and a negative correlation between fasting plasma leptin concentrations and obesity (Pannacciulli et al., 2006), suggesting leptin mediated role of the putamen in regulating food intake. Dysconnectivity between the putamen and PG in obesity could be associated through the mechanism for leptin regulation.

The hippocampus also showed inverse association in its connectivity with the PG, while the hippocampi showed anti-correlation with the PG in the one-sample *T*-test. It is implicated that the anti-correlation is positively associated with BMI. Whilst the hippocampi also receive dopaminergic afferent from the ventral tegmental area, the association between hippocampus and food intake manipulation as well as physical exercise has been well documented. Physical exercise, which is shown to prevent excessive weight gain, acutely increases hippocampal volume, while hippocampal reduction is found after a series of cafeteria-diet in mice (Sack et al., 2017). Calorie restriction has been shown to improve cognition by upregulating brain-derived neurotrophic factor (BDNF) and downregulating oxidative stress in the hippocampus (Kishi et al., 2015). The hippocampus has been shown to be activated when images of high-calorie food are presented and this activation has been found to be a function of fasting plasma levels of insulin (Wallner-Liebmann et al., 2012), suggesting that the hippocampi respond to energy stimuli under the influence of hunger status. Our PG-hippocampus dysconnectivity finding may implicate that the PG is regulated by the hippocampus based on the hunger status and food availability.

Several limitations of the current study need to be addressed. First, as this study is based on functional connectivity, where functional connectivity is estimated by co-activations of two regions, causal relationships are not illuminated, although it could be inferred through known endocrinological properties of the PG. Second, despite the known subdivisions within the PG, such as the anterior and posterior pituitary, we avoided to make distinctions due to the imaging resolution of 2 mm^3^ voxels and smoothing employed in processing. These two regions that showed functional dysconnectivity associated with BMI may arise from two distinct sub-regions of the PG.

It needs to be also addressed that the current study does not make distinction whether the PG connectivity influences BMI or BMI influences PG connectivity. While brain connectivity has been shown to influence body weight specifically in the context of the reward system, body weight status induced by surgical procedures has also been shown to influence functional brain connectivity (Lepping et al., 2015). It remains unclear whether our connectivity findings are the cause or results of the body weight.

In this study, we found functional dysconnectivity between the PG and dopaminergic regions including the putamen, hippocampus, and OFC. The results implicate dopaminergic modulation between the PG and these regions that influences body weight.

## Ethics Statement

This study was approved by the University of Mississippi Institutional Review Board. This study had no direct involvement of human or animal subjects. All human subjects gave written informed consent.

## Author Contributions

PR and TI designed the study and drafted the manuscript. TI analyzed the data.

## Conflict of Interest Statement

TI has been a consultant for Sumitomo Dainippon and received speaker’s honoraria from Eli Lilly and Daiichi Sankyo, and Dainippon Sumitomo. The remaining author declares that the research was conducted in the absence of any commercial or financial relationships that could be construed as a potential conflict of interest.
